# Cutting down the time to identify challenging tumor therapeutic targets and drug combinations using synthetic lethal approaches

**DOI:** 10.12688/f1000research.13679.1

**Published:** 2018-03-12

**Authors:** John S. Lazo

**Affiliations:** 1 Departments of Pharmacology and Chemistry, Fiske Drug Discovery Laboratory, University of Virginia, Box 800735, Charlottesville, VA, 22908-0735, USA

**Keywords:** Synthetic lethality, drug combinations, anticancer drugs, screening, synergy

## Abstract

Cancer drug discoverers and developers are blessed and cursed with a plethora of drug targets in the tumor cells themselves and the surrounding stromal elements. This bounty of targets has, at least in part, inspired the rapid increase in the number of clinically available small-molecule, biological, and cellular therapies for solid and hematological malignancies. Among the most challenging questions in cancer therapeutics, especially for small molecules, is how to approach loss-of-function gene mutations or deletions that encode tumor suppressors. A second mounting question is what are the optimal drug combinations. This article will briefly review the recent advances in exploiting *in vitro* and *in vivo* synthetic lethal screens to expose cancer pharmacological targets with the goal of developing new drug combinations.

## Introduction

**Figure 1.**
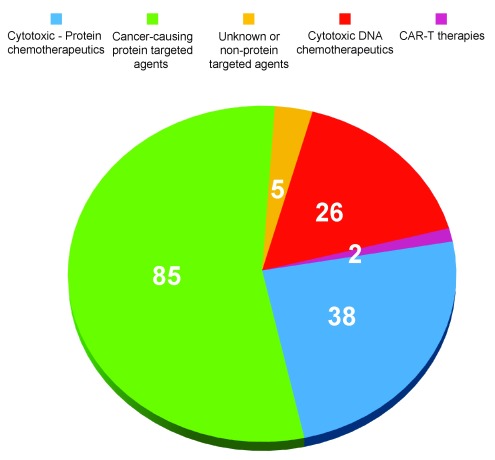
Mechanistic distribution of current US Food and Drug Administration-approved anticancer drugs. The classification scheme in this figure was inspired by Santos *et al*. ^3^. CAR–T, chimeric antigen receptor–T cell.

More than six decades of concerted laboratory and clinical labor have enabled us to control and even cure some cancers ^1, 2^. Nonetheless, these successes represent the minority, and many tumors either are innately resistant or become resistant to the available therapies. We know that human cancer is complex with vast numbers of genetic alterations and it remains a major cause of human morbidity and death. In large part, we still fail to understand why some cancers are responsive to certain drugs whereas other tumors are not. This issue has become even more complicated because there has been an enormous increase in the number of US Food and Drug Administration (FDA)-approved treatments for cancer. Last year, Santos *et al*. ^3^ divided the 154 FDA-approved cancer drugs broadly into four groups: 26 drugs are cytotoxic agents, such as those that disrupt DNA synthesis or integrity; 38 drugs are broadly cytotoxic and act at least partially through protein targets, such as the proteasome or microtubules; 85 drugs mechanistically target cancer-associated proteins; and 5 drugs act through unknown or non-protein targets. One should add to this list the two recent FDA approvals for chimeric antigen receptor–T cell (CAR–T) therapy to make a total of 156 agents ( Figure 1). Combining multiple drugs to treat cancer is now standard clinical practice, but the actual components of the drug combinations often were generated empirically. The growth in the numbers of anticancer drugs makes this process much more challenging because of the number of possible theoretical combinations. Moreover, it is now believed that there are at least 600 cancer drivers across different cancers and there is enormous heterogeneity in the genetic composition of cells, even in a single tumor from a patient, not to mention among the metastases. Furthermore, tumor cells even in the same tumor exist in diverse environmental conditions, including differences in nutrient availability, pH, cytokine concentrations, oxygen levels, and reactive oxygen species quantities. This enormous complexity portends the development of even more anticancer agents ^2^.

Among the 600 cancer drivers, many, such as tumor suppressors, have been considered to be beyond the reach of traditional therapeutic intervention, but this concept is now being challenged ^1^. This report examines one experimental approach that has recently become popular as a potential means to expose new druggable targets and to accelerate the identification of rational drug combinations: synthetic lethality screening. This somewhat mechanistically agnostic methodology enables one to uncover previously unknown cooperative interactions that sustain viability or another therapeutically relevant phenotype between the product of two genes, two compounds, or a compound and a gene product.

## Theoretical approaches to discovering cancer drug combination synergy

The concept of enhancing a therapeutic effect by combining different independent agents is ancient. Originally, combinations were purely observationally driven by perceptive and persistent medical practitioners. The principle of combination chemotherapy is currently most widely practiced and advanced in cancer. Historically, clinicians avoided using drugs that had overlapping organ toxicities and tried to apply drugs that had different molecular targets to reduce the potential for drug resistance. In the second half of the previous century, we developed a fairly good understanding about the biochemical reactions required for DNA synthesis, which enabled the creation of antimetabolites and antifolates, many of which we still use today. Thoughtful studies by Sartorelli *et al*. ^4, 5^, Darnowski and Handschumacher ^6^, and Damon and Cadman ^7^ emphasized the importance of targeting the rate-limiting steps in the biochemical reactions and identifying the optimal sequence of drug exposure. Mayer and Janoff ^8^ illustrated the potential importance of maintaining constant ratios of the individual drugs whenever possible in the tumor and elsewhere.

The development of rigorous methods to quantify drug synergism has been an important area of early pharmacological and toxicological studies within and outside the area of cancer. One of the earliest published attempts to distinguish true compound synergy from additivity was published by Bliss ^9^, who built his toxicological methodology on the pioneering mathematical work of J. H. Gaddum ^10^. Since then, there have been multiple attempts to provide a universally accepted quantitative definition of drug synergy, but these have had only limited success. Chou highlighted the fact that as many as 13 different methods for determining synergism exist in the literature with considerable disagreement among them ^11^. Reasons for the discrepancies stem from differing mechanistic assumptions, variable compound ratios, and the shapes of the individual drugs’ concentration response or dose response curves. A synergistic effect is not simply a result that is greater than the arithmetic sum of the effects of two drugs alone. For example, if a drug has a very steep dose response curve and one combines a minimally effective dose of compound A (a dose that caused a 1% effect or an ED _1_) with another minimally effective dose (ED _1_) of compound A, it is possible that an effect greater than an ED _2_ will be observed. This does not, however, indicate that compound A is acting synergistically with itself. A Cartesian plot of the relative effects of the individual compounds can provide an isobologram, which allows one to distinguish between additivity or synergy ^12^. In cancer, one is generally interested in selective tumor cell killing, but many of the cytotoxic agents have low therapeutic indices and also damage non-malignant cells that are not involved in tumor growth or dissemination. Moreover, one should be striving for a large change rather than small changes in tumor viability, even with targeted therapies, if there is any hope for a meaningful survival advantage ^13^. The mass-action law-based median-effect method has become one method that has been prominently employed in cancer studies ^11, 14, 15^. Unfortunately, earlier attempts to mathematically define synergy or antagonism, including the median-effect method, assume idealized mass-action principles and employ logarithmic linearization data analyses, which can lead to poor model fitting ^16, 17^. Non-linear regression has been suggested to be a superior and more robust methodology to determine synergy ^16, 17^.

## Empirical approaches to discovering cancer drug combination synergy with synthetic lethality studies

The fundamental concept of synthetic lethality generally has been attributed to the genetic studies conducted by Calvin Bridges with *Drosophila melanogaster* a century ago ^18^. The actual term “synthetic lethality”, however, was coined 20 years later by Theodore Dobzhansky to describe the combination of two genetic events that created something new, in this case lethality, but only when both genes were lost ^19^. It took almost half a century for cancer biologists and pharmacologists to embrace the idea of exploiting synthetic lethality to identify new drug combinations, initially using yeast and human cancer cells ^20, 21^. The growth of synthetic lethal screening and cancer has been reviewed elsewhere ^20– 22^. One popular pharmacologically oriented synthetic lethal cancer screening approach uses the expression of a gain-of-function proto-oncogene, such as an activated kinase, and evaluates a library of drugs seeking sensitization against a phenotypic endpoint, such as proliferation or death ^22^. Alternatively, RNA interference (RNAi) or, more recently, clustered regularly interspaced short palindromic repeats (CRISPR)-based methods have been used to suppress the expression of a druggable gene product in cancer cells that are then treated with compounds from chemical libraries or RNAi libraries. Another strategy is to expose cancer cells to two different drugs and search for synergistic alterations in the phenotypic endpoint. Historically, gain-of-function mutations were viewed as much more druggable than loss-of-function mutations where restoration by adding a small molecule would be the goal ^1^. Excitement for synthetic lethality studies was further stimulated by the concept of tumor cell “addiction” to oncogenes, formulated by the late Bernard Weinstein ^23^, by which he meant cell death induced by disabling a required oncoprotein. Although the mechanistic basis for the so-called “addiction” was not well formulated, it had functional appeal for many investigators. The rapid realization that there was “addiction” even to non-oncogenic proteins opened up an even larger array of possible targets ^24^. The use of large-scale small interfering RNA and short hairpin RNA screens revealed not only gain-of-function mutations that could be therapeutically approached yet also more challenging but, perhaps more interesting, loss-of-function mutations or deletions that are possible synthetic lethality participants ^25, 26^. Some of these synthetic lethality leads fell victim to the common pitfalls that often occur during preclinical cancer target validation, including the well-known off-target effects of small interfering RNA and short hairpin RNA ^13^. Nonetheless, in 2014, the first drug identified by using synthetic lethality screen received FDA approval: the poly(ADP-ribose) polymerase (PARP) inhibitor olaparib for the treatment of germline BRCA-mutated advanced ovarian cancer, which has cells that are DNA damage repair-deficient ^27, 28^. The use of the PARP inhibitor has now been extended to maintenance treatment for patients with recurrent epithelial ovarian, fallopian tube, or primary peritoneal cancer who are having partial or complete responses to platinum-based chemotherapy. Thus, systematic mapping of disease drivers by using a synthetic letha lapproach can provide future therapeutic strategies by both identifying potential targets and highlighting key pathways that can be drugged.

## The future of cancer drug combination studies

In my opinion, the genetic and epigenetic heterogeneity and complexity within and between tumors are likely to dictate the continued exploitation of empirical approaches to discovering cancer drug combinations. Nonetheless, a recent publication provides an interesting window into how the future of drug combination studies might evolve ^29^. The rapidly expanding genome editing tools, such as CRISPR-Cas9 ^30– 32^, provide powerful platforms, which can even be used in preclinical animal models. Manguso *et al*. ^29^ used a pooled *in vivo* CRISPR-Cas9 strategy with transplantable B16 melanoma tumors injected into mice and then treated the mice with a monoclonal antibody against the PD-1 checkpoint and identified previously unknown genes that sensitized the tumors to the PD-1 immunotherapy *in vivo*. In particular, the authors found that suppression of the protein tyrosine phosphatase *Ptpn2* increased the efficacy of anti-PD-1 therapy by enhancing interferon-gamma-mediated effects on antigen presentation and growth suppression. Although protein tyrosine phosphatases have historically been labeled “undruggable” at least with small molecules ^1^, there have been several recent examples of successful drugging of this protein class ^33– 36^. This should encourage others to follow.

I expect that *in vivo* genomic editing screens will have a major role in defining the future of anticancer drug combinations. Some of the issues that exist with the current genomic editing methodology, however, are the permanence of the DNA changes; the quantal nature of the silencing; off-target endonuclease activity associated with the use of Cas9; and the possibility for transcriptional, translational, or post-translational compensation during the gene deletion steps. More transient editing methods that target RNA might help resolve concerns about the quantal and permanent nature of expression. Human tumors often have cells with amplified gene-encoding regions containing oncogenes or heterozygous loss of genes encoding tumor suppressors, which are challenging to emulate using current genomic editing technology. The timing or scheduling of the individual drug combinations could also have important consequences with respect to tumor response, and more reversible, concentration-controllable, methods could allow one to determine the optimal drug sequence and doses. Of course, the consequences of removing a protein are not always reproduced by the simple binding of a small molecule to the protein unless one is using methods, such as proteolysis targeting chimeras ^37^, to deplete intracellular proteins. Another matter that is not easily addressed with any of the existing genomic editing methods is how to design combinations with multiple drugs.

Genetic studies have demonstrated that some synthetic lethal interactions are contextually dependent and cell type-specific ^22^. For synthetic lethal interactions to be successfully transferred from laboratory models to humans, the contextual parameters will need to be carefully outlined in both fundamental and clinical studies. In the absence of well-defined and measurable conditions, a more general pan-synthetic lethal interaction might be more desirable than one that targets the so-called private interactions ^22^. However, it should be noted that recent studies suggest that drug additivity or synergy may not be universally necessary for a positive benefit with drug combinations ^38^.

Even with these limitations, there is considerable enthusiasm for the use of synthetic lethal strategies to accelerate the identification of anticancer drug combinations. Moreover, there appears to be no reason to believe that the synthetic lethality approach would not be generally useful for designing treatments for other non-malignant diseases. Nevertheless, a critical question remains as to whether synthetic lethal strategies, regardless of how they are designed, will actually accelerate the drugging of challenging targets or expose new clinically productive anticancer drug combinations. As we have learned repeatedly, cancer is a complex collection of diseases and appealing methodologies often do not live up to our expectations.

## Abbreviations

CRISPR, clustered regularly interspaced short palindromic repeats; FDA, US Food and Drug Administration; PARP, poly(ADP-ribose) polymerase; RNAi, RNA interference.

## References

[1] Lazo JS Sharlow ER: Drugging Undruggable Molecular Cancer Targets. *Annu Rev Pharmacol Toxicol.* 2016;56:23–40. 10.1146/annurev-pharmtox-010715-103440 26527069

[2] Workman P Al-Lazikani B: Drugging cancer genomes. *Nat Rev Drug Discov.* 2013;12(12):889–90. 10.1038/nrd4184 24287764

[3] Santos R Ursu O Gaulton A: A comprehensive map of molecular drug targets. *Nat Rev Drug Discov.* 2017;16(1):19–34. 10.1038/nrd.2016.230 27910877 PMC6314433

[4] Sartorelli AC: Some approaches to the therapeutic exploitation of metabolic sites of vulnerability of neoplastic cells. *Cancer Res.* 1969;29(12):2292–9. 4905235

[5] Sartorelli AC Shansky CW Rosman M: Biochemical approaches to the combination chemotherapy of colon cancer. *Cancer.* 1975;36(6 Suppl):2445–8. 10.1002/1097-0142(197512)36:6<2445::AID-CNCR2820360628>3.0.CO;2-A 764962

[6] Darnowski JW Handschumacher RE: Enhancement of fluorouracil therapy by the manipulation of tissue uridine pools. *Pharmacol Ther.* 1989;41(1–2):381–92. 10.1016/0163-7258(89)90115-0 2652156

[7] Damon LE Cadman EC: The metabolic basis for combination chemotherapy. *Pharmacol Ther.* 1988;38(1):73–127. 10.1016/0163-7258(88)90103-9 3293090

[8] Mayer LD Janoff AS: Optimizing combination chemotherapy by controlling drug ratios. *Mol Interv.* 2007;7(4):216–23. 10.1124/mi.7.4.8 17827442

[9] BLISS CI: THE TOXICITY OF POISONS APPLIED JOINTLY. *Annals of Applied Biology.* 1939;26:585–615. 10.1111/j.1744-7348.1939.tb06990.x

[10] Gaddum J: Reports on biological standards. III. Methods of biological assay depending on a quantal response. *Spec Rep Ser Med Res Council.*London1933; (183). Reference Source

[11] Chou TC: Drug combination studies and their synergy quantification using the Chou-Talalay method. *Cancer Res.* 2010;70(2):440–6. 10.1158/0008-5472.CAN-09-1947 20068163

[12] Tallarida RJ: Drug synergism: its detection and applications. *J Pharmacol Exp Ther.* 2001;298(3):865–72. 11504778

[13] Kaelin WG: Common pitfalls in preclinical cancer target validation. *Nat Rev Cancer.* 2017;17(7):425–40. 10.1038/nrc.2017.32 28524181

[14] Holbeck SL Camalier R Crowell JA: The National Cancer Institute ALMANAC: A Comprehensive Screening Resource for the Detection of Anticancer Drug Pairs with Enhanced Therapeutic Activity. *Cancer Res.* 2017;77(13):3564–76. 10.1158/0008-5472.CAN-17-0489 28446463 PMC5499996

[15] Mathews Griner LA Guha R Shinn P: High-throughput combinatorial screening identifies drugs that cooperate with ibrutinib to kill activated B-cell-like diffuse large B-cell lymphoma cells. *Proc Natl Acad Sci U S A.* 2014;111(6):2349–54. 10.1073/pnas.1311846111 24469833 PMC3926026

[16] Ashton JC: Drug combination studies and their synergy quantification using the Chou-Talalay method--letter. *Cancer Res.* 2015;75(11):2400. 10.1158/0008-5472.CAN-14-3763 25977339

[17] Bozic I Reiter JG Allen B: Evolutionary dynamics of cancer in response to targeted combination therapy. *eLife.* 2013;2:e00747. 10.7554/eLife.00747 23805382 PMC3691570

[18] Bridges CB: The Origin of Variations in Sexual and Sex-Limited Characters. *Am Nat.* 1922;56(642):51–63. 10.1086/279847

[19] Dobzhansky T: Genetics of natural populations; recombination and variability in populations of Drosophila pseudoobscura. *Genetics.* 1946;31:269–90. 20985721 10.1093/genetics/31.3.269 PMC1209328

[20] Hartwell LH Szankasi P Roberts CJ: Integrating genetic approaches into the discovery of anticancer drugs. *Science.* 1997;278(5340):1064–8. 10.1126/science.278.5340.1064 9353181

[21] Nijman SM: Synthetic lethality: general principles, utility and detection using genetic screens in human cells. *FEBS Lett.* 2011;585(1):1–6. 10.1016/j.febslet.2010.11.024 21094158 PMC3018572

[22] O'Neil NJ Bailey ML Hieter P: Synthetic lethality and cancer. *Nat Rev Genet.* 2017;18(10):613–23. 10.1038/nrg.2017.47 28649135

[23] Weinstein IB: Cancer. Addiction to oncogenes--the Achilles heal of cancer. *Science.* 2002;297(5578):63–4. 10.1126/science.1073096 12098689

[24] Luo J Solimini NL Elledge SJ: Principles of cancer therapy: oncogene and non-oncogene addiction. *Cell.* 2009;136(5):823–37. 10.1016/j.cell.2009.02.024 19269363 PMC2894612

[25] Mullard A: Synthetic lethality screens point the way to new cancer drug targets. *Nat Rev Drug Discov.* 2017;16(9):589–91. 10.1038/nrd.2017.165 28860588

[26] Zhao D Depinho RA: Synthetic essentiality: Targeting tumor suppressor deficiencies in cancer. *Bioessays.* 2017;39(8). 10.1002/bies.201700076 28675450 PMC8610080

[27] Lord CJ Ashworth A: PARP inhibitors: Synthetic lethality in the clinic. *Science.* 2017;355(6330):1152–8. 10.1126/science.aam7344 28302823 PMC6175050

[28] Lord CJ Tutt AN Ashworth A: Synthetic lethality and cancer therapy: lessons learned from the development of PARP inhibitors. *Annu Rev Med.* 2015;66:455–70. 10.1146/annurev-med-050913-022545 25341009

[29] Manguso RT Pope HW Zimmer MD: *In vivo* CRISPR screening identifies *Ptpn*2 as a cancer immunotherapy target. *Nature.* 2017;547(7664):413–8. 10.1038/nature23270 28723893 PMC5924693

[30] Han K Jeng EE Hess GT: Synergistic drug combinations for cancer identified in a CRISPR screen for pairwise genetic interactions. *Nat Biotechnol.* 2017;35(5):463–74. 10.1038/nbt.3834 28319085 PMC5557292

[31] Shen JP Zhao D Sasik R: Combinatorial CRISPR-Cas9 screens for *de novo* mapping of genetic interactions. *Nat Methods.* 2017;14(6):573–6. 10.1038/nmeth.4225 28319113 PMC5449203

[32] Wang T Yu H Hughes NW: Gene Essentiality Profiling Reveals Gene Networks and Synthetic Lethal Interactions with Oncogenic Ras. *Cell.* 2017;168(5):890–903.e15. 10.1016/j.cell.2017.01.013 28162770 PMC5445660

[33] Chen YN LaMarche MJ Chan HM: Allosteric inhibition of SHP2 phosphatase inhibits cancers driven by receptor tyrosine kinases. *Nature.* 2016;535(7610):148–52. 10.1038/nature18621 27362227

[34] Yu ZH Zhang ZY: Regulatory Mechanisms and Novel Therapeutic Targeting Strategies for Protein Tyrosine Phosphatases. *Chem Rev.* 2018;118(3):1069–1091. 10.1021/acs.chemrev.7b00105 28541680 PMC5812791

[35] Stanford SM Bottini N: Targeting Tyrosine Phosphatases: Time to End the Stigma. *Trends Pharmacol Sci.* 2017;38(6):524–40. 10.1016/j.tips.2017.03.004 28412041 PMC5494996

[36] Bai Y Yu ZH Liu S: Novel Anticancer Agents Based on Targeting the Trimer Interface of the PRL Phosphatase. *Cancer Res.* 2016;76(16):4805–15. 10.1158/0008-5472.CAN-15-2323 27325652 PMC4987244

[37] Burslem GM Crews CM: Small-Molecule Modulation of Protein Homeostasis. *Chem Rev.* 2017;117(17):11269–301. 10.1021/acs.chemrev.7b00077 28777566

[38] Palmer AC Sorger PK: Combination Cancer Therapy Can Confer Benefit via Patient-to-Patient Variability without Drug Additivity or Synergy. *Cell.* 2017;171(17):1678–1691.e13. 10.1016/j.cell.2017.11.009 29245013 PMC5741091

